# The Peru Approach against the COVID-19 Infodemic: Insights and Strategies

**DOI:** 10.4269/ajtmh.20-0536

**Published:** 2020-06-04

**Authors:** Aldo Alvarez-Risco, Christian R. Mejia, Jaime Delgado-Zegarra, Shyla Del-Aguila-Arcentales, Arturo A. Arce-Esquivel, Mario J. Valladares-Garrido, Mauricio Rosas del Portal, León F. Villegas, Walter H. Curioso, M. Chandra Sekar, Jaime A. Yáñez

**Affiliations:** 1 Carrera de Negocios Internacionales, Facultad de Ciencias Empresariales y Economicas, Universidad de Lima, Lima, Peru;; 2 Universidad Continental, Lima, Peru;; 3 Facultad de Ciencias Administrativas y Recursos Humanos, Instituto de Consumo, Universidad de San Martin de Porres, Lima, Peru;; 4 Universidad Nacional de la Amazonia Peruana, Iquitos, Peru;; 5 Escuela Nacional de Marina Mercante “Almirante Miguel Grau,” Callao, Peru;; 6 Department of Health and Kinesiology, The University of Texas at Tyler, Tyler, Texas;; 7 Universidad Nacional Agraria La Molina, Lima, Perú;; 8 Facultad de Ciencias y Filosofia, Universidad Peruana Cayetano Heredia, Lima, Peru;; 9 College of Pharmacy, University of Findlay, Findlay, Ohio;; 10 Facultad de Educacion, Carrera de Educacion y Gestion del Aprendizaje, Universidad Peruana de Ciencias Aplicadas, Lima, Peru;; 11 Gerencia Corporativa de Asuntos Científicos y Regulatorios, Teoma Global, Lima, Peru

## Abstract

The COVID-19 epidemic has spawned an “infodemic,” with excessive and unfounded information that hinders an appropriate public health response. This perspective describes a selection of COVID-19 fake news that originated in Peru and the government’s response to this information. Unlike other countries, Peru was relatively successful in controlling the infodemic possibly because of the implementation of prison sentences for persons who created and shared fake news. We believe that similar actions by other countries in collaboration with social media companies may offer a solution to the infodemic problem.

## INTRODUCTION

Peru is facing a tremendous burden from the COVID-19 pandemic, as it is among the top 15 countries in the world in reported COVID-19 cases and second in Latin America, only after Brazil.^1^ On May 25, 2020, Peru reported 123,979 COVID-19 cases with a 2.9% lethality rate.^2^ Presently, with more than 60 days of lockdown and social isolation in Peru, the COVID-19 crisis is expected to markedly affect people’s well-being, as has been reported elsewhere.^3–6^

The COVID-19 crisis is reported to cause increased anxiety^7,8^ as people’s work and normal life are disrupted, causing an unprecedented impact on mental health.^9,10^ This disruption has been accompanied by an infodemic of fake news, as reported by the World Health Organization (WHO) on February 15, 2020.^11^ Sylvie Briand, architect of the WHO’s strategy to counter the infodemic, observed that misinformation and false reports spread faster because of social media.^11^ To counteract this, the WHO provides up-to-date information via its social media and website and urges people to act appropriately.^11^

The fight against the infodemic is a real challenge, as it spreads very rapidly on social media. The infodemic has been accompanied by reports of racism and discrimination against Chinese nationals and patients in the United Kingdom^12^ and a surge of unproven religious and herbal treatments for COVID-19 prevention in India.^13^ Unproven prescription drugs have been falsely promoted for COVID-19 prevention and treatment, including hydroxychloroquine plus azithromycin, tocilizumab, or ivermectin.^14^ Caretas, a magazine in Peru, portrayed ivermectin as a potential treatment on its front page, which outraged the scientific and medical community because it promoted self-medication.^15^

### Possible cause of the infodemic: low health literacy and free time.

**Figure 1.**
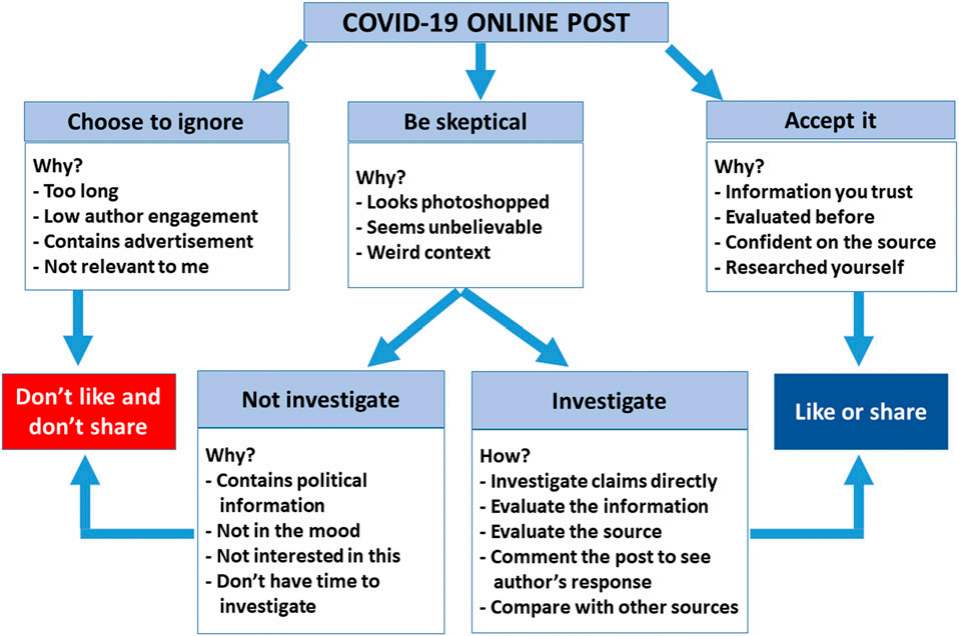
Decision tree and factors to consider before liking or sharing a COVID-19 online post or ignoring it. Figure adapted from McQuate.^19^

In a population with a low literacy rate, increased availability of free time as result of the COVID-19 lockdown may have contributed to the infodemic. Health literacy has been defined as the individuals’ capacity to obtain, process, and understand basic health information and services needed to make appropriate health decisions,^16^ and to address or solve a health-related problem.^17^ Reports show that a rumor has a three times greater chance to be shared on social media than a verified story,^18^ and lack of health literary in a population will have the capability to amplify the problem. Figure 1 shows a decision tree on how to address a COVID-19 online post.^19,20^ The general recommendation is to look for alerts including the length of the post, extent of engagement, whether it contains advertisements, and its relevance to the reader. The only information one should share is information that has been investigated and is understood by the reader.

### Peruvian government response against the COVID-19 infodemic.

We present as examples three of the most relevant fake news items that were denied by the Peruvian government, using official social media.

1. Death forecast: “125,000 people could die in Peru”^21^ (dated: March 19, 2020). This was a personal commentary by a news reporter in Peru and was rapidly shared on social media. His forecast was based on the number of confirmed cases during the first 10 days of the pandemic, an exponential increase, and a worst case scenario with absence of social isolation. However, Peru had already implemented strict social isolation measures and a nationwide lockdown. The forecast raised alarm in the population and was wrong, as the death toll as of May 25, 2020 in Peru is 3,629^2^.
2. Rumors about the minister of economy contracting COVID-19 based on “visible symptoms”^16^ (dated: March 11, 2020). These rumors were based on a photograph on Twitter.^16^ It was a malicious post because a photograph is not a diagnostic tool.
3. When Martin Vizcarra, the president of Peru, did not host his daily press conference, it started a rumor that he was very sick and hospitalized because of COVID-19. (dated: March 29, 2020). This was denied by the Ministry of Health of Peru via its Twitter account the same day,^22^ and on May 19 during his press conference, President Vizcarra informed that to stop those ongoing rumors, he was tested and his result was negative.^23^

### Implemented strategies in Peru against the COVID-19 infodemic.

The reported fake news in March were summarized by the “Handbook Covid-19 Peru” website.^24^ This is similar in concept to other international websites such as Salud sin Bulos,^25^ Salud con Lupa,^26^ and Maldita.es,^27^ which have so far been able to identify more than 500 global hoaxes related to COVID-19. The last entry in the Handbook Covid-19 Peru is dated March 27, 2020.^24^ The reason for this was possibly the announcement via Twitter^28^ on April 8 by the Ministry of Justice and Human Rights of Peru that persons who share fake news and misinform others to obtain a benefit or to perturb the public tranquility can be sanctioned with a prison sentence. The Ministry urged people to share only official information, accompanied by the hashtag “Don’t Spread #FakeNews.”^28^ It further indicated that those who create and/or share false information to benefit themselves or cause perjury to others will receive a 2- to 4-year prison sentence, and if the fake news causes panic and perturbs the public tranquility, the sentence may be 3–6 years.^28,29^ Peru became the first country in Latin America to implement prison sentences for creating and disseminating fake news.

This measure was widely applauded, as it appeared to result in a dramatic drop in fake news accounts. The term fake news was again mentioned in Peru^23^ only on May 19, when the President announced that to deny rumors, he was tested for COVID-19.

### Other measures to combat the COVID-19 infodemic.

Efforts by social media and other technology companies to curb the infodemic have now been implemented. Twitter proposed to curb the spread of COVID-19 fake news by deleting accounts that spread fake news.^30^ Similar efforts have been made by ebay and Amazon^31–33^ by deleting offers of products marketed as miracle cures.

There is a proposal to include health preventive measures into elementary and high school curricula.^34^ It has been reported that the promotion of health literacy in schools resulted in enhanced basic cancer literacy among middle and high school students.^35^

Health agencies have been urged to have a more proactive and agile public health presence on social media and to combat the spread of fake news by directly responding to fake news and by building disease detection and surveillance systems through social media and unusual activity monitoring.^36^ The U.K. National Health Service (NHS) website describes specific actions against COVID-19 online fake news.^37^ Similarly, the Pan American Health Organization website provides risk communication guidelines for fake news.^38^

Artificial intelligence approaches, such as a deep convolutional neural network (FNDNet), for the automatic detection of COVID-19 fake news showed 98% accuracy.^39^ In similar terms, the U.K. NHS joined forces with Google, Twitter, Instagram, and Facebook to provide accurate information about COVID-19 and to prevent the spread of fake news.^40^

## CONCLUSION

The COVID-19 outbreak has highlighted the need to target infodemics that can be as detrimental as an actual epidemic. It will be a multifactorial fight because we will need to increase health literacy in the population, establish a stronger presence of national health agencies in social media, develop better detection tools, and enable action by governments, as Peru has implemented. More research should go into the use of artificial intelligence to better respond to the ever-increasing spread of fake news via social media. However, we all need to remember that it is in our hands to share or not to share, before truly verifying such information. Like all news, fake news has an author, most of the times unknown. Let us be sure that we are not the authors of such news.
